# Criminal Responsibility

**Published:** 1895-12

**Authors:** T. D. Crothers

**Affiliations:** Hartford, Conn.


### Correspondence.

### CRIMINAL RESPONSIBILITY.

*Editor Buffalo Medical Journal :*

SIR—My attention has been called to Dr. Putnam's very clearly stated paper on The Criminal Plea of Irresponsibility, in the November number of the JOURNAL. The attempt to mark out boundary lines of responsibility in crime, and determine where the law should punish and where it should excuse, always ends in confusion and obscuration of the real facts. Questions of criminal responsibility are very largely theories and personal opinions, hence the difference of opinions. This is to be expected from judges and lawyers, but reflects sadly on the physician, who should have the special training, and certainly has the facilities, for ascertaining the facts and conditions from which criminality springs. No theories of courts or legal definitions of insanity should have any influence with physicians, unless founded on tangible facts that can be sustained by evidence accessible to every medical man. Many good physicians still cling to the medieval conceptions that crime and insanity has a moral basis which can be reached by exorcism, physical punishment and the infliction of pain to the higher mentality, rousing it to greater control of the lower. Along the line of such theories originate the struggle to find some conditions or mental states which are to be treated by punishment and otherwise. It is always sad to hear physicians refer to the decisions of judges and the findings of jurors in certain cases, as if they were absolute truths and conclusions of the highest wisdom and science. In reality, along lines of mental disease and questions of science, they are not unfrequently the most stupid blunders and misconceptions. All medical men, familiar with the facts of mental disease, find such wide departures from the truth in theories and rulings of the law as to make agreement impossible. Dr. Putnam's suggestion, that I have asserted that one glass of spirits would produce a permanent impression on the brain, and that anyone who had ever taken alcohol was not responsible, is such an extreme statement as to require no denial. I am pleased to say that for many years I have urged the irresponsibility of criminal inebriates, entirely from a clinical and physical point of view. It is no statement of theory, but a question of facts and their meaning. I have never seen an inebriate criminal who was not suffering from disease which could be defined and traced as clearly as any other form of insanity. My observations and conclusions have been confirmed by an ever-increasing number of expert medical men, both at home and abroad. The question is purely one of facts. If inebriety, or drunkenness, or states of intoxication are not insanities in the broadest sense, what are they? And what is the physical condition of the brain in respect to the capacity for healthy functional activity in these states? Is it possible for anyone to repeatedly, or at intervals, paralise the brain and nervous system with alcohol, and be of sound and healthy mind? Let the answer to this come from a scientific study of cases, not from theories or opinions of courts and judges.

The present rulings of the law and courts fail, to a large degree, to represent the advances of science, and in many instances notoriously fail in the punishment of inebriates who commit crime, fail practically and ethically, and follow lines of precedent built up entirely on theories that are contradicted flatly by an ever-increasing array of facts. As physicians we can have no quarrel with the law, but, when it becomes an interpreter of science, we should demand that it recognise the facts above all theories or opinions. The danger is along the line of theories and assumption. The medical man should be a gleaner and interpreter of facts alone, no matter what the relation these facts may have to law, or theory or public opinion. If inebriates are irresponsible legally, this fact can have no possible danger to society or civilisation. It is the theories and errors of the criminal responsibility of inebriates, which are unsupported by facts, that are dangerous. It is the personal equation and theoretical conceptions of inebriety which appear in strange conclusions that are to be feared. We need a thorough study of cases, above all theories, to determine the questions of accountability and irresponsibility in criminal inebriates. Until this is done, there will be difference of opinion and confusion of practice.

HARTFORD, CONN. T. D. CROTHERS, M. D.

